# Gene Order Phylogeny and the Evolution of Methanogens

**DOI:** 10.1371/journal.pone.0006069

**Published:** 2009-06-29

**Authors:** Haiwei Luo, Zhiyi Sun, William Arndt, Jian Shi, Robert Friedman, Jijun Tang

**Affiliations:** 1 Department of Biological Sciences, University of South Carolina, Columbia, South Carolina, United States of America; 2 The Graduate Program in Organismic and Evolutionary Biology, University of Massachusetts, Amherst, Massachusetts, United States of America; 3 Department of Computer Science and Engineering, University of South Carolina, Columbia, South Carolina, United States of America; University of California San Diego, United States of America

## Abstract

Methanogens are a phylogenetically diverse group belonging to Euryarchaeota. Previously, phylogenetic approaches using large datasets revealed that methanogens can be grouped into two classes, “Class I” and “Class II”. However, some deep relationships were not resolved. For instance, the monophyly of “Class I” methanogens, which consist of Methanopyrales, Methanobacteriales and Methanococcales, is disputable due to weak statistical support. In this study, we use MSOAR to identify common orthologous genes from eight methanogen species and a Thermococcale species (outgroup), and apply GRAPPA and FastME to compute distance-based gene order phylogeny. The gene order phylogeny supports two classes of methanogens, but it differs from the original classification of methanogens by placing Methanopyrales and Methanobacteriales together with Methanosarcinales in Class II rather than with Methanococcales. This study suggests a new classification scheme for methanogens. In addition, it indicates that gene order phylogeny can complement traditional sequence-based methods in addressing taxonomic questions for deep relationships.

## Introduction

Methanogens play an important role in the global carbon cycle by producing methane [1]. They are phylogenetically widespread within the Phylum Euryarchaeota. Five Orders of methanogens have been identified: Methanopyrales, Methanococcales, Methanobacteriales, Methanomicrobiales and Methanosarcinales [2]. There are three pathways of biological methane production: the hydrogenotrophic pathway, the aceticlastic pathway, and the methylotrophic pathway. The hydrogenotrophic pathway is found in all methanogens, while the other two pathways are limited to Methanosarcinales [3]. The universal distribution of hydrogenotrophic pathway suggests that the hydrogenotrophic methanogenesis may be the ancestral form of biological methane production and that hydrogenotrophic methanogenesis may appear only once during evolution [3].

Phylogenetic analyses, using concatenations of translation and transcription-related proteins or other universally distributed proteins with conserved functions, revealed that methanogens can be grouped into two classes [3]. However, the deep relationships within methanogens were not resolved. For instance, it has been proposed that Methanopyrales, Methanococcales, and Methanobacteriales form a monophyletic clade, but this clade failed to gain reliable statistical support [3], [4].

Comparisons of bacterial genomes from different species revealed that gene order is not conserved. Gene order has proven to be a useful phylogenetic character to resolve species relationships, such as the phylogenetic reconstruction of mitochondrion and chloroplast genomes [5], [6], [7] and bacterial genomes [8], [9]. Alteration of gene order in unichromosomal genomes is achieved via inversion, transposition, and inverted transposition [10], [11], and it is believed that such events are rare during evolution. Hence, it is likely that gene order data can be used to resolve deep phylogenetic relationships [11]. In this study, we reconstruct distance-based gene order phylogeny to resolve the ancient relationships of methanogens.

## Results and Discussion

### Orthologs shared among 8 methanogen and Pyrococcus furiosus genomes

Valid reconstruction of the gene order phylogeny depends on accurate identification of shared orthologous genes. Because the nine genomes of interest are highly diverged and their genome sizes vary from 1.69 Mbp to 5.75 Mbp, a relatively small number of shared orthologous genes are expected. On the other hand, many gene rearrangement events are expected to be observed between these genomes because of their remote relation and wide distribution over five Classes (evolutionary units), indicating that more identifiable shared genes will be preferable for the purpose of reconstructing the gene order phylogeny. Also, we found that the loss of a small proportion of orthologs could cause significant loss of the phylogenetic signals (data not shown). Therefore, procedures for recovery of orthologous genes solely based upon sequence similarities were not able to retrieve a complete and accurate set of shared orthologous genes for this study.

**Figure 1:**
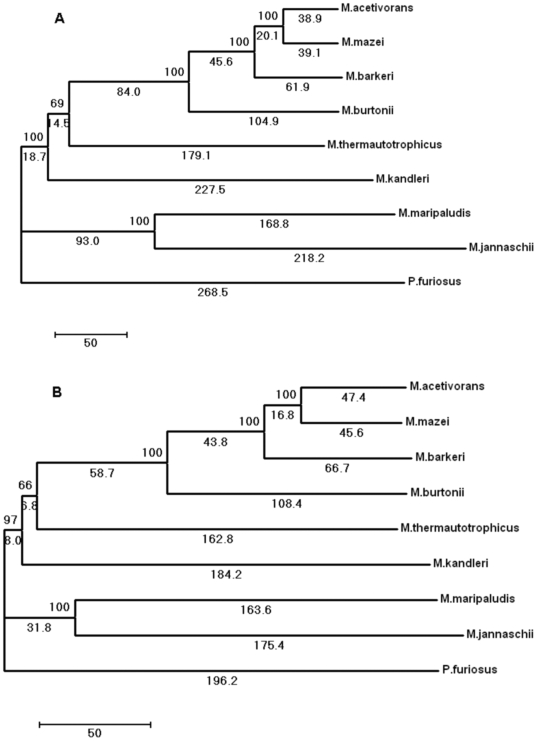
Phylogeny of eight methanogenic genomes inferred from (A) an empirically derived estimator (EDE) distance and (B) a breakpoint distance matrix. Values at nodes show the number of times that the clade defined by that node appears in the 100 jackknife trees. Values under branches and the scale bar show the number of genome rearrangement events. Pyrococcus furiosus was used as an outgroup.

MSOAR software identifies orthologous genes by using information on both sequence similarity and genome rearrangement. Therefore, it has the potential to identify more true orthologs than methods based solely on sequence comparisons. MSOAR revealed 477 orthologous genes shared by all eight methanogens and the outgroup *Pyrococcus furiosus* genomes. Although some methanogen species are subject to frequent HGT events [4], including a distant outgroup species (*Pyrococcus furiosus*) can reduce possible xenologs (alien copy due to HGT) in the identified ortholog groups. We then used the genomic positional information of these 477 shared orthologs to reconstruct the gene order phylogeny. The high statistical support (Fig. 1A & 1B) in gene order trees obtained by using jackknife resampling technique (50% removal of 477 common orthologs) suggests that the shared 477 orthologs carry a robust phylogenetic signal.

### Sequence-based phylogeny of methanogens

**Figure 2:**
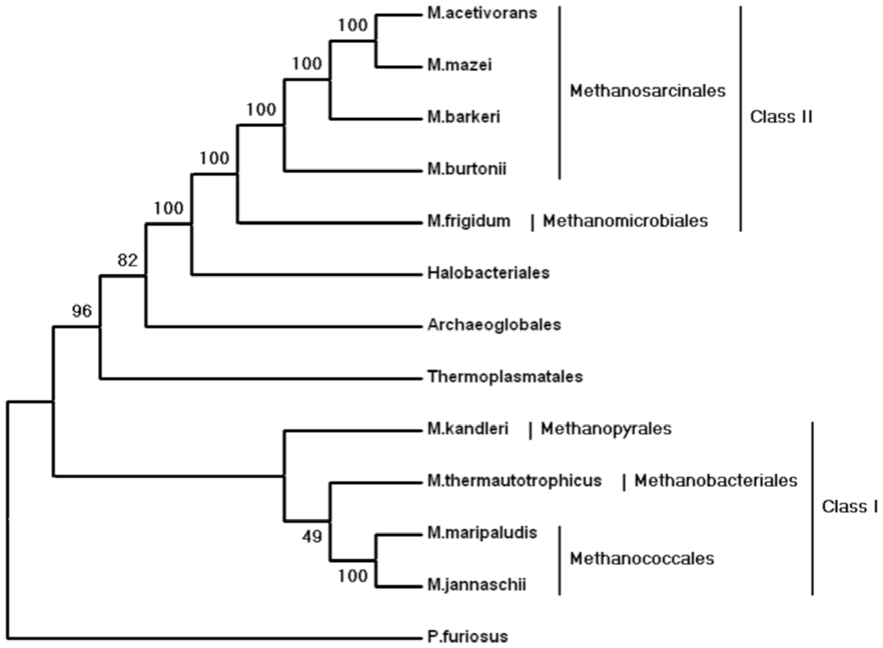
Phylogeny of methanogens inferred from fusion analyses of 53 ribosomal proteins. The tree was reconstructed using a maximum likelihood method. Two classes of methanogens were proposed. The tree is a reprint from Bapteste et al [3]. The intervening nonmethanogenic archaeal species are represented using the name of Order they belong to. Only bootstrap values .45% are indicated.

The methanogenic archaeal phylogeny was previously reconstructed using a concatenation method from the translation and transcription-related proteins [3], [12] and from a set of 31 universally distributed proteins involving a broad range of functions [4]. The phylogeny showed that methanogens are not a monophyletic group, and two classes of methanogens were proposed [3]. Class I includes Methanopyrales, Methanobacteriales, and Methanococcales, and Class II consists of Methanosarcinales and Methanomicrobiales [3], [4]. While the monophyly of the Class II methanogen was supported by a strong bootstrap value, the Class I methanogen phylogeny lacked sufficient statistical support to be considered as a monophyletic group (Fig. 2). The phylogenetic analysis of concatenated alignments of 31 universally distributed proteins also provided weak support for Class I [4].

It was thought that a phylogenetic approach using a large dataset would capture sufficient signal to resolve phylogenetic relationships, provided that xenologs from horizontal gene transfer (HGT) events are adequately identified [13]. For instance, one study claimed that the core genome of the Gamma-Proteobacteria was identified free of HGT and could be used to reconstruct a robust phylogeny of Gamma-Proteobacteria [14]. Another study proposed that the “Tree of Life” may be resolved by concatenation of 31 orthologs occurring in 191 species [15]. However, further analyses demonstrated that it cannot be determined whether a large portion of the genes in the concatenation method have a common ancestry [16], [17]. Even by using a set of 22 carefully aligned core genes, each of which displays topological congruence and branch-length congruence and has a similar phylogenetic signal, the deep nodes of prokaryotic phylogeny were poorly resolved [18]. Furthermore, between-species phylogenetic analyses indicated that orthologous replacement is quite common in the evolution of prokaryotes, even occurring on widely distributed and functionally conserved genes [16]. Orthologous replacement is the substitution of the native gene with an alien copy, either by homology-dependent recombination or through introduction of the alien one and subsequent loss of the original gene [16]. Both scenarios obliterate the phylogenetic signal in gene trees and concatenated alignment-based species trees [16].

It is widely accepted the “informational” genes whose transcripts involve translation, transcription, and replication are much less prone to HGT than “operational” genes encoding for metabolic enzymes, transport systems, and signal transduction related enzymes [19]. This serves the rationale for reconstruction of a ribosomal protein tree for methanogens. However, other studies demonstrated that “informational” genes including those encoding for ribosomal proteins are subject to HGT [19], [20]. In fact, there are no dramatic differences in the rates of HGT between informational and operational genes [19], [21]. HGT may explain weak support for Class I methanogens using concatenated sequences of translation and transcription-related proteins as well as those widely distributed and functional conserved proteins [3], [4]. Hence, the monophyly of Class I methanogen needs to be reevaluated.

### Gene order phylogeny of methanogens

There are at least three ways that breakpoints can occur in a unichromosomal genome, inversion, transposition, and inverted transposition. In some prokaryotic genomes, HGT events are common. One scenario in HGT, acquisition of a gene copy at one genomic position followed by loss of the original copy at another position [16], can be erroneously treated as transposition or inverted transposition, depending on whether the alien copy is on the same strand as the original copy. It is difficult to trace the breakpoints contributed by transposition and inverted transposition. However, a transposition is equivalent to three inversions, and an inverted transposition is equivalent to two inversions [22]. To simplify computation, an altered gene order can be considered to have resulted from a series of inversion events. Hence, the distance matrix for the gene order phylogeny reconstruction of unichromosomal genomes is usually generated by computing breakpoint and inversion distances [8], [9]. Breakpoint distance measures the number of gene adjacencies occurring in one genome but absent from the other genome, hence breakpoint distance describes the dissimilarity of the gene order between two genomes [22]. Inversion distance is computed through the minimum number of inversion events that are required to convert one genome to the other [22]. Simulation studies have shown that both breakpoint and inversion distances may underestimate the true evolutionary distance. Therefore, a distance-correction algorithm, the empirically derived estimator (EDE), can improve the distance estimates [22]. Since gene rearrangements are rare events, gene order phylogeny can resolve deep relationships [22]. For instance, gene order data has been used to resolve 30 species relationships within the Class Gamma-Proteobacteria [8]. However, no studies have used gene order data to resolve more ancient relationships. In this study, we use gene order data to analyze deep relationships covering multiple Classes within the Phylum Euryarchaeota.

Using gene order data to analyze eight methagen genomes, we generated both an inversion distance-based tree and a breakpoint distance-based tree that are both consistent and congruent (Fig. 1A & 1B). The gene order tree has many nodes in common with the phylogenetic tree that was derived from the concatenated sequence of 53 ribosomal proteins [3]. For instance, they have the same branching pattern for Methanosarcinales and Methanococcales (Fig. 2). In addition, the gene order phylogeny also grouped the methanogens into two classes. However, the gene order tree strongly suggests a different branching pattern from that of sequence concatenation-based phylogenetic tree. In the gene order tree, Methanopyrales and Methanobacteriales clustered together with Methanosarcinales with high statistical support (Fig. 1). In contrast, in the sequence-based tree, they group with Methanococcales known as “Class I” with weak support (Fig. 2).

### Limitations of gene order phylogeny

Important questions remain to be answered whether methanogens comprise a monophyletic group and whether hydrogenotrophic methanogenesis arose once during evolution. If the methanogens are a monophyletic clade, then hydrogenotrophic methanogenesis could have arisen once during evolution. If not, there are two possible scenarios, *i.e.* either hydrogenotrophic methanogenesis evolved multiple times or it appeared once and was lost in other non-methanogenic lineages within the clade. Alternatively, hydrogenotrophic methanogenesis could have evolved once and been transferred to other lineages by HGT. Although the evidence using sequence concatenation-based phylogenetic approaches does not support monophyly and maintains that Halobacteriales, Thermoplasmatales, and Archaeoglobales are positioned between “Class I” and “Class II” methanogens (Fig. 2) [3], [4], [12], a recent phylogenetic study using a conditioned reconstruction algorithm shows methanogens form a monophyletic clade in the archaeal tree [23].

We cannot position Halobacteriales, Thermoplasmatales, and Archaeoglobales in the gene order phylogeny of methanogens, since inclusion of these organisms significantly obliterates the phylogenetic signals. A possible reason could be that inclusion of more genomes decrease the number of shared orthologous genes, which results in degradation of phylogenetic signals. In this case, gene order-based phylogenetic analysis cannot test whether methanogens are a monophyletic clade, and sequence-based approaches are more useful, though they sometimes produce contradictory results.

Overall, our result suggests that gene order phylogeny can complement the traditional sequence-based methods in addressing taxonomic questions and resolving ancient relationships.

## Materials and Methods

### Genome annotation

To date, 19 methanogenic archaeal genomes have been sequenced and assembled. The 19 methanogens fall into four Classes in the Phylum *Euryarchaeota*. In this study, we analyzed eight representative methanogen genomes that span the four Classes of methanogens and were previously analyzed by sequence substitution-based phylogenetic approaches [3], [12]. These eight species include *Methanococcus maripaludis* C5 (CP000609), *Methanocaldococcus jannaschii* DSM 2661 (L77117), *Methanothermobacter thermautotrophicus* str. Delta H (AE000666), *Methanococcoides burtonii* DSM 6242 (CP000300), *Methanosarcina barkeri* str. Fusaro (CP000099), *Methanosarcina mazei* Go1 (AE008384), *Methanosarcina acetivorans* C2A (AE010299), and *Methanopyrus kandleri* AV19 (AE009439). We notice that the *Methanogenium frigidum*, one species in the Order Methanomicrobiales, was represented in sequence-based tree [3], but its whole genomic sequence is currently unavailable for gene order analysis. The whole genomic DNA sequences of the eight methanogen genomes and the outgroup genome *Pyrococcus furiosus* DSM 3638 (AE009950) were downloaded from NCBI and annotated by the RAST Server [24]. The RAST Server provides a fully automated annotation for bacterial and archaeal genomes using subsystem technology in a Genbank file format [24]. Using Perl scripts, this file was parsed for the predicted protein-coding gene transcripts and their corresponding genomic positions as well as strandedness.

### Ortholog identification

Each pair of genomes was processed with a reciprocal all-versus-all BLASTP search with an E-cutoff value of 0.1 [25]. The output file was formatted and combined with information on gene location and strandedness. Then the MSOAR software [26], [27] was used to identify common orthologs in a pair of genomes. MSOAR is a high-throughput genome-scale ortholog assignment system. It is a two-step procedure where homologous genes are first identified by a sequence similarity search and then paralogous genes are differentiated from the orthologs by comparison of the genome context of each gene [26], [27]. We select a genome as a reference genome, which can be any of the nine genomes. MSOAR was then used to identify shared orthologs between the reference genome and each of the remaining eight genomes. Afterwards, the pairwise ortholog sets were used to identify the common ortholog sets occurring in the nine archaeal genomes.

### Gene order generation and gene order phylogeny reconstruction

The genomic positions of all protein-coding regions were extracted by Perl scripts. The order of orthologs in each genome was determined based upon their starting position and strandedness. GRAPPA [11], [28] was used to compute the pairwise inversion and breakpoint distances from the gene order data and output distance matrices. Then the inversion and breakpoint distance-based phylogenetic trees were reconstructed by FastME software [29] and visualized by MEGA4 [30]. To calculate the statistical reliability of the tree branches, we applied a jackknife resampling technique, which randomly removed 50% of the initial orthologous gene sets while retaining the relative order of the remaining genes [9]. We generated 100 jackknife random samples, and the CONSENSE program in the PHYLIP software package [31] was used to obtain a majority-rule consensus tree with the numbers at each node representing the percentage that the clade defined by that node appears in the 100 jackknife trees.
